# Deciphering Ancestral Sex Chromosome Turnovers Based on Analysis of Male Mutation Bias

**DOI:** 10.1093/gbe/evz221

**Published:** 2019-10-12

**Authors:** Armando Acosta, Mónica L Martínez-Pacheco, Karina Díaz-Barba, Niccole Porras, Mariana Gutiérrez-Mariscal, Diego Cortez

**Affiliations:** 1 Center for Genome Sciences, UNAM, Cuernavaca, Mexico; 2 Biotechnology Institute, UNAM, Cuernavaca, Mexico

**Keywords:** synonymous substitution rates, sex chromosomes, sex chromosome system turnovers, male mutation bias

## Abstract

The age of sex chromosomes is commonly obtained by comparing the substitution rates of XY gametologs. Coupled with phylogenetic reconstructions, one can refine the origin of a sex chromosome system relative to specific speciation events. However, these approaches are insufficient to determine the presence and duration of ancestral sex chromosome systems that were lost in some species. In this study, we worked with genomic and transcriptomic data from mammals and squamates and analyzed the effect of male mutation bias on X-linked sequences in these groups. We searched for signatures indicating whether monotremes shared the same sex chromosomes with placental mammals or whether pleurodonts and acrodonts had a common ancestral sex chromosome system. Our analyses indicate that platypus did not share the XY chromosomes with placental mammals, in agreement with previous work. In contrast, analyses of agamids showed that this lineage maintained the pleurodont XY chromosomes for several million years. We performed multiple simulations using different strengths of male mutation bias to confirm the results. Overall, our work shows that variations in substitution rates due to male mutation bias could be applied to uncover signatures of ancestral sex chromosome systems.

## Introduction

Current evolutionary models recognize that sex chromosomes evolved from a pair of autosomes following the emergence of a sex-determining gene and the arrest of homologous recombination around this locus (Bachtrog 2013). The synonymous substitution rates of XY (or ZW) gametologs, together with specific speciation events, are used to trace the origin of sex chromosomes. A previous study applied this approach to date the origin of sex chromosomes in therian and monotreme mammals (Cortez et al. 2014). The XY chromosomes in placental mammals and the Y_5_X_5_ sex chromosomes in monotremes originated ∼181–175 Ma. However, another study (Lahn and Page 1999) suggested that the therian XY chromosomes originated 300 Ma, and thus, monotremes might have maintained the placental sex chromosomes for a period of time before their split from placental mammals (167–192 Ma; data retrieved from Timetree database; http://www.timetree.org/; last accessed October 16, 2019) and the origin of the current Y_5_X_5_ chromosomes.

Contrary to mammals, reptiles show both old and recent sex chromosomes (Tree of Sex 2014; Gamble et al. 2015; Sabath et al. 2016; Pennell et al. 2018). For example, pleurodonts, anguimorpha, and many snakes have conserved their sex chromosomes for >100 Myr (Marin et al. 2017; Altmanova et al. 2018; Augstenova et al. 2018; Rovatsos et al. 2019), whereas boas and chameleons exhibit recently acquired sex chromosomes (<50 Myr old) (Rovatsos et al. 2015; Gamble et al. 2017; Augstenova et al. 2018). Furthermore, transitions between sex determination systems show a higher frequency in reptiles compared to mammals, birds, and amphibians (Pennell et al. 2018).

**Fig. 1.**
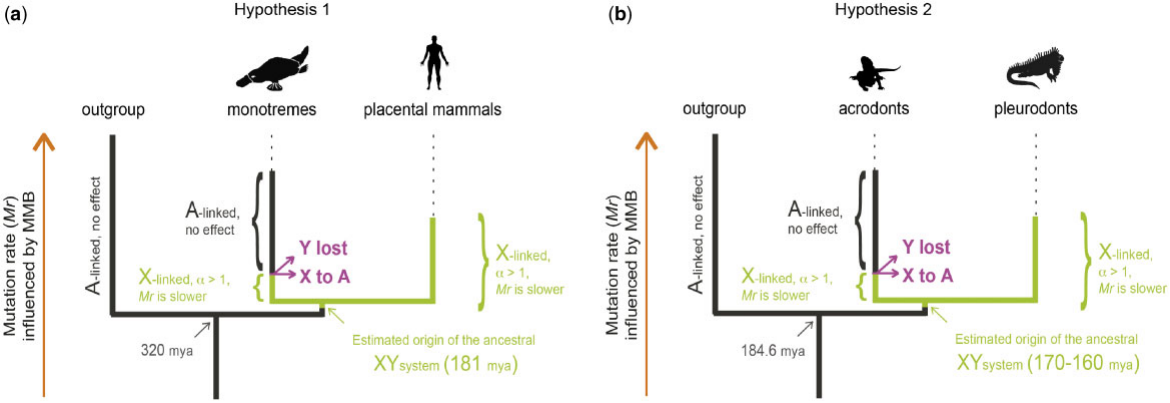
—The hypotheses tested in the study. (*a*) In the first hypothesis, the XY chromosomes present in placental mammals were shared by monotremes during an unknown amount of time before they were replaced by the current X_5_–Y_5_ chromosomes (Ezaz et al. 2005). (*b*) In the second hypothesis, acrodonts and pleurodonts shared the same XY chromosomes during an unknown amount of time before they were replaced in acrodonts by other sex determination systems. In both hypotheses, synonymous substitution rates of X-linked sequences were differentially affected by male mutation bias, whereas synonymous substitution rates of orthologous sequences in outgroup species, located on autosomes (A-linked), were not differentially affected by male mutation bias. In platypus and acrodonts, synonymous substitution rates of former X-linked sequences were only differentially affected by male mutation bias during the time they remained X-linked. Once the ancestral XY chromosome systems were lost, the X chromosomes became autosomes and their synonymous substitution rates were no longer differentially affected by male mutation bias.

Additionally, a recent study dated the XY chromosome system in the green anole, *Anolis carolinensis*, which originated 170–160 Ma (Marin et al. 2017). The sex chromosomes arose around the time pleurodonts and acrodonts diverged (Zheng and Wiens 2016). In the pleurodont clade (i.e., iguanas, anoles, spiny lizards, etc.) most species share the same pair of XY chromosomes (Rovatsos, Pokorna et al. 2014; Altmanova et al. 2018). Moreover, ancestral state reconstructions of squamate sex determination systems indicate that the sex determination system in the ancestor of pleurodonts and acrodonts (i.e., agamids and chameleons) was probably temperature-dependent (Gamble et al. 2015). However, phylogenetic analyses of the oldest XY gametologs from two pleurodont species, *A. carolinensis* and the Fiji-banded iguana (*Brachylophus fasciatus*), showed that the XY chromosomes could have originated prior to the divergence of pleurodonts and acrodonts (Marin et al. 2017). This result is relevant since extant species of agamids and chameleons show either a great variety of sex chromosomes or thermally induced sex-biased offspring (Tree of Sex 2014). These results suggest that acrodonts may have undergone an ancestral sex determination system turnover that consisted in the loss of the pleurodont XY chromosomes (fig. 1). Pleurodonts and acrodonts offer a unique opportunity to test methods to estimate the age of ancestral sex chromosomal losses, which, for the moment, can only be indirectly inferred using phylogenetic-based analyses.

In species with XY chromosomes, sex-linked sequences have distinctive mutation rates compared to autosomal sequences (Wilson Sayres and Makova 2011). In numerous vertebrates, male gametes go through a higher number of replication cycles than female gametes, leading to a male-biased mutation rate linked to replication errors. This phenomenon is known as male mutation bias (MMB) and its strength is reflected in the values taken by α; α is defined as the ratio of male to female mutation rate. Hence, when MMB is present in a given lineage, α values are >1 (Drost and Lee 1995; Hurst and Ellegren 1998; Makova and Li 2002). MMB was first described in rodents and primates (Wolfe and Sharp 1993; McVean and Hurst 1997; Makova and Li 2002; Sandstedt and Tucker 2005; Venn et al. 2014) and later observed in most mammals (Wilson Sayres et al. 2011; Link et al. 2017), birds (Bartosch-Harlid et al. 2003), snakes (Vicoso et al. 2013), and fish (Ellegren and Fridolfsson 2003).

When MMB is present (α > 1), the male-specific Y chromosome is expected to be the fastest evolving chromosome, followed by the autosomes, which spend half their time in males and a half in females. Finally, the X chromosome, which spends only one-third of its time in males is expected to be the slowest evolving chromosome (Wilson Sayres and Makova 2011). In species with ZW chromosomes, the expected pattern is that Z-linked sequences would evolve faster than W-linked and autosomal sequences (Wilson Sayres and Makova 2011). The strength of α is relative to the general mutation rate in the germ-line and the generation time of the species (Amster and Sella 2016). MMB has a larger effect in species with extended lifespans and late sexual maturity such as primates (Amster and Sella 2016). Nevertheless, in theory, one could use the effect of MMB on the mutation rates of ancient sex-linked sequences, and exploit this information to trace ancestral sex chromosomal losses.

In this study, we show that the analysis of MMB is useful to find signatures of ancestral sex chromosomes. We tested our approach under two scenarios. We first hypothesized that monotreme mammals (platypus) shared the XY chromosomes with placental mammals for a period of time (fig. 1*a*). We then tested a second hypothesis in which agamids maintained the pleurodont XY chromosomes for a number of million years (fig. 1*b*). In this second hypothesis, we specifically tested whether hypothetical former X-linked sequences in three agamid species (*Pogona vitticeps*, *Ctenophorus decresii*, and *Phrynocephalus vlangalii*) showed traces of MMB. Presently, these three agamid species appear to harbor three different sex determination systems: *P. vitticeps* shows ZW microsex chromosomes (Ezaz et al. 2005) as well as thermally induced sex reversal (Li et al. 2016), *C. decresii* shows temperature-dependent sex determination (Harlow 2000) and finally, *Phr.vlangalii* has differentiated ZW chromosomes (Zeng et al. 1997).

In both hypotheses (fig. 1*a* and *b*), when the ancestral XY chromosomes were replaced by a new sex chromosomal system, the Y chromosome disappeared from the population, whereas the X chromosome was restored as an autosome (fig. 1*a* and *b*). However, owing to MMB, the mutation rate on former X-linked sequences in agamids and monotremes decreased during the time these species conserved the ancestral XY chromosome system (fig. 1*a* and *b*). In contrast, X-linked sequences in pleurodonts and therians have been differentially affected by MMB since the origin of the XY chromosome system, whereas orthologous sequences from outgroup species should not show traces of MMB (fig. 1*a* and *b*).

We found that platypus does not show signatures of sharing the sex chromosomes with placental mammals. Furthermore, we found that pleurodont species probably exhibit a strong MMB (α = 6). Consistently, former X-linked sequences in three agamid species showed substitution rates that are higher than the substitution rates from three pleurodont species but lower than the substitution rates from five snake species. These observations suggest that agamids conserved the ancestral XY chromosome system for several million years. Numerous simulations confirmed the results and helped establish that the *Pogona/Ctenophorus* and *Phrynocephalus* lineages conserved the ancestral XY chromosomes for 110–160 and 80–100 Myr, respectively. Our method will surely be refined as we learn more about the germ-line mutation rates in pleurodonts and we obtain more accurate α values.

## Materials and Methods

### Data Collection

Mammalian species were selected based on two criteria: having the maximum number of one-to-one orthologous genes to the human X genes and defined male mutation bias. The threshold was set to 144 one-to-one orthologous genes. CDS sequences for human, rat, mouse, rabbit, dolphin, dog, elephant, and armadillo, as well as CDS sequences from outgroup species, opossum, platypus, chicken, Chinese soft-shell turtle, and *Xenopus*, were obtained from the ENSEMBL database (release 90; www.ensembl.org; last accessed October 16, 2019). For reptiles, our aim was to analyze the potential sex chromosome transition in acrodonts. We searched in public databases for genomic and transcriptomic data for acrodont species and their sister groups: pleurodonts, anguimorpha, and snakes. The species were selected based on the maximum number of X-linked genes (and orthologous sequences) with complete or almost complete CDS. The threshold was set to 77 one-to-one orthologous genes. Lack of pleurodont species with sufficient genomic or transcriptomic data, besides *A. carolinensis*, persuaded us to produce dedicated data for the study. We generated strand-specific RNA-seq libraries (using the Illumina TruSeq Stranded mRNA Library protocol) for five tissues (brain, heart, liver, kidney, and ovaries) from a female green iguana (*Iguana iguana*) and five tissues (brain, heart, liver, kidney, and ovaries) from a female leopard-lizard (*Gambelia wislizenii*); BioProject accession number PRJNA490698. Both individuals were sacrificed using a lethal dose of pentobarbital; this study met legal regulations and institutional procedures for the investigation of the University of Mexico. The project was reviewed and approved by the Ethical Committee of the University (approval project No. 369). RNA quality was assessed using a Fragment Analyzer machine from Advanced Analytical. Each library was sequenced on Illumina HiSeq 2000 platform at the Massive Sequencing Unity of the University of Mexico (UNAM). At least 17 million sequencing reads (100-nt, paired-end) were produced for each library. The female green iguana and the female leopard-lizard were bought from authorized pet shops. We downloaded the complete chromosomal sequences and coding sequences (CDS) from the reference genomes of *A. carolinensis* available on the ENSEMBL database (release 90; www.ensembl.org; last accessed October 16, 2019). We downloaded the CDS sequences for *P. vitticeps* from the Pogona Genome Project (Georges et al. 2015). Similarly, we downloaded from the SRA database (www.ncbi.nlm.nih.gov/sra; last accessed October 16, 2019) RNA-seq data for *C. decresii*, *Phr.vlangalii* (Yang et al. 2014), *Deinagkistrodon acutus* (Yin et al. 2016), and *Echis coloratus*, *Pantherophis guttatus*, and *Opheodrys aestivus* (Hargreaves et al. 2014). RNA-seq data for *Thamnophis elegans* were downloaded from the Reptilian Transcriptomes v2.0 database (Tzika et al. 2015). Transcriptomic sequences were produced using an Illumina Hiseq 2000 or Hiseq 2500 machines and showed a minimum of 20 million reads of 100-nt long. Additionally, we collected the genomic sequences for a male *A. carolinensis* and a male Fiji-banded iguana (*B. fasciatus*) from ref. (Marin et al. 2017). Libraries were DNA-seq, sequenced on Illumina HiSeq 2500 sequencers (100-nt paired-end reads).

### Assessing Chromosomal Substitution Rates at X-, Y-Linked, and Autosomal Intronic Sequences from *A. carolinensis* and *B. fasciatus*

We estimated X-linked, Y-linked, and autosomal substitution rates as proxies of mutation rates. We worked with intronic sequences from the green anole, *A. carolinensis*, and the Fiji-banded iguana (*B. fasciatus*). We applied an approach we had previously used in mammals (Link et al. 2017) where we compared aligned sequences between two species to calculate the chromosomal substitution rates. Briefly, we assembled the genomic raw reads from the Fiji-banded iguana into scaffolds with SOAP-de novo (Luo et al. 2012) (kmer = 31). We selected all scaffolds in the *A. carolinensis* reference genome and Fiji-banded iguana assemblies that mapped using BlastN (Altschul et al. 1990) to known Y- and X-linked genes in *A. carolinensis*; others and us have previously identified the Y-linked genes (Marin et al. 2017) and X-linked genes in this species (Alfoldi et al. 2011; Rovatsos, Altmanova et al. 2014; Marin et al. 2017). To limit the risk of including nonorthologous positions in the alignments, we considered only the intronic sequences that were located in the same scaffolds as conserved one-to-one orthologous exons in *A. carolinensis* and the Fiji-banded iguana. Then, we used Lagan20 (Brudno et al. 2003), an alignment program designed to work on noncoding sequences, to align the concatenated exonic and intronic sequences from *A. carolinensis* and the Fiji-banded iguana. We then removed ambiguous positions using Gblocks (Talavera and Castresana 2007) and excluded the first intron of all genes from the alignments because these introns often contain various regulatory elements (Chamary and Hurst 2004). We further removed potentially hidden exons from the alignments and the first 20 intronic nucleotides flanking the exons in order to remove regulatory sites such as splicing sites and splicing enhancers (Pink and Hurst 2010). We also removed all detectable C_p_G sites from our alignments because C_p_G sites could cloud the effect of male mutation bias (Taylor et al. 2006). Finally, only those genes showing intronic alignments >1,000 bp were considered for the analyses since shorter sequences could produce more extreme values. In order to minimize uncertainties in the substitution rate estimates of X- and Y-linked sequences, we applied a nonparametric double bootstrap approach (Axelsson et al. 2004), which bootstraps the intronic alignments by both introns and sites. We repeated this procedure 1,000 times, and for each new alignment, we calculated the substitution rate using the Tamura–Nei model with the baseml program, implemented in the PAML44 package (Yang 1997).

The α value for pleurodonts species was calculated using the median values of X, Y, and autosomal substitution rates and Miyata’s three equations (Miyata et al. 1987): 1) α(Y/X) = 2/[(3X/Y)−1], 2) α(Y/A) = 1/[(2A/Y)−1], and 3) α(X/A) = [4−(3X/A)]/[(3X/A)−2].

### Assessing Chromosomal Synonymous Substitution Rates at X-Linked and One-to-One Orthologous Genes

We downloaded from the ENSEBML database (release 90; www.ensembl.org; last accessed October 16, 2019) the lists of one-to-one orthologous genes across *A. carolinensis*, human, mouse, elephant, opossum, platypus, chicken, the Chinese soft-shell turtle, and *Xenopus*, and selected only the orthologous genes to the X-linked genes in *A. carolinensis* (X-linked genes found in Marin et al. 2017). RNA-seq reads for five snakes (*D. acutus*, *E*. *coloratus*, *Pantherophisguttatus*, *O*. *aestivus*, and *T. elegans*), three agamids (*P. vitticeps*, *C. decresii*, and *Phr.vlangalii*) and two pleurodonts (*I. iguana* and *G. wislizenii*) were assembled using Trinity (v2.0.2) (Grabherr et al. 2011) and one-to-one orthologous sequences to the *A. carolinensis* X-linked genes were identified using best-bidirectional BlastN (Altschul et al. 1990) searches.

X genes from different strata have the potential to affect the substitution rates since genes found in younger strata or in the pseudoautosomal regions should show a similar mode of inheritance to the autosomes. Therefore, we focused the analyses on genes located on scaffolds of the X chromosome that in *A. carolinensis* exhibit only one copy in males (data taken from Alfoldi et al. 2011; Rovatsos, Altmanova et al. 2014; Marin et al. 2017). Although detailed information about chromosomal strata or pseudoautosomal regions on the X chromosome of pleurodonts is not known, the Y chromosomes in this group has highly degenerated since it conserves only 2% of its original gene content (Marin et al. 2017), and analyses of various X-linked genes in many families of pleurodonts consistently show amplifications corresponding to a single copy in males (Rovatsos, Pokorna et al. 2014; Altmanova et al. 2018); except for corytophanids. The information available, therefore, suggests that the majority of X-linked genes in pleurodonts have likely not recombined with the Y chromosomes for >110 Myr (estimated divergence time between the two studied species: *A. carolinenesis* and *B. fasciatus*), and most likely, many of them stopped recombination when the sex chromosomes originated 170–160 Ma (Marin et al. 2017).

Next, we built codon-based concatenated and individual alignments using PRANK (Loytynoja and Goldman 2005) for the 77 X-linked genes in *A. carolinensis* with one-to-one orthologous sequences in all other 17 species of amniotes (supplementary table 1, Supplementary Material online). We then performed a bootstrapping approach by sampling random codons from the concatenated alignment until we obtained the same number of sites as in the original concatenate. We repeated this procedure 100 times, and for each new concatenated alignment, we calculated a *d*_S_ tree using the codeml program, implemented in the PAML44 package (Yang 1997). This bootstrap approach was used to reduce potential biases introduced by individual genes and/or positions. We obtained a consensus *d*_S_ tree by taking the median values for each branch from the 100 individual (bootstrapped) *d*_S_ trees. We repeated this procedure 100 times for autosomal genes in *A. carolinensis* and their one-to-one orthologous sequences in the other 17 species of amniotes. For each round, we randomly selected 77 autosomal genes from the reference genome, aligned the nucleotide sequences of the selected genes, and constructed a *d*_S_ tree. In order to obtain comparable estimates for snakes, agamids, and pleurodonts, we added all branch lengths from the individual species’ branch length until the ancestral branch length corresponding to the ancestor of snakes or the ancestor of agamids/pleurodonts; thus, each species’ estimate would be composed of *d*_S_ values representing the same amount of time (from the last common ancestor of snakes/pleurodonts until the present). Next, in order to correct for potential biases introduced by fast or slow evolving groups, we corrected the X-linked synonymous substitution rates using averaged autosomal synonymous substitution rates, which served as proxies of the overall genomic mutation rate in each lineage. We worked with the *d*_S_ tree’s branch lengths as proxies of synonymous substitution rates. So, for snakes, agamids, and pleurodonts, we used the added branch lengths (from the last common ancestor of snakes/pleurodonts until the branches of the individual species) of the autosomal consensus tree to calculate the differences across median values of squamates species. We used the differences across the species’ individual medians and the average value of all species from the autosomal consensus *d*_S_ tree in order to correct the branch lengths for pleurodonts, agamids, and snakes in the X-linked consensus tree. For example, if a given lineage presented a long branch in the autosomal consensus *d*_S_ tree (i.e., a lineage with high substitution rate), the difference with the average value of all species in the autosomal consensus *d*_S_ tree would result in a large positive number and, therefore, we would reduce the species’ branch length in the X-linked tree by a larger value. On the other hand, if a given lineage presented a particularly short branch in the autosomal consensus *d*_S_ tree (i.e., a lineage with low substitution rate), the difference with the average value of all species in the autosomal consensus *d*_S_ tree would result in large negative number and, therefore, we would increase the species’ branch length in the X-linked tree by a larger value. We only corrected the branches of snakes, agamid, and pleurodonts. In order to further reduce the uncertainties within groups, we averaged the estimates of snakes and pleurodonts, separately. We repeated the autosomal-based correction using 100 random sets of autosomal sequences as a substitute for the X-linked sequences. We reckon that the complete gene set of the ZW chromosomes in *Pogona* and *Phrynocephalus* is not known and some genes we considered as autosomal could be sex-linked in these species. However, we expect that the average signal from hundreds of randomly selected genes will reflect the average autosomal rate rather than a rate from a minority of sex-linked genes. Moreover, since chromosome 6 from *A. carolinensis* is orthologous to the Z chromosome in snakes (Vicoso et al. 2013) genes from this chromosome were avoided in the analyses.

We downloaded from the ENSEBML database (release 90; www.ensembl.org; last accessed October 16, 2019) the lists of one-to-one orthologous genes across human, rat, rabbit, dolphin, dog, elephant, armadillo, platypus, chicken, *A. carolinensis*, and *Xenopus*, and selected only the orthologous genes to the X-linked genes in human. For all selected species MMB was previously estimated (Wilson Sayres et al. 2011). We worked with 114 X-linked genes in human that are one-to-one orthologous in all other amniote species (supplementary table 1, Supplementary Material online). We reconstructed both the individual *d*_S_ trees for the 114 X-linked genes and the concatenated *d*_S_ tree. We worked with the oldest genes on the X chromosome. That is, X-linked genes from the oldest strata (X-conserved region; Pandey et al. 2013) were considered for the analyses. Most of these genes stopped recombination with the Y chromosome 180 Ma when the sex chromosomes originated (Cortez et al. 2014). We built codon-based alignments using PRANK (Loytynoja and Goldman 2005) and performed the individual and bootstrapped sampling of random codons from the concatenated alignment 100 times using the codeml program, implemented in the PAML44 package (Yang 1997). We also selected random subsets of 104 autosomal genes to obtain the overall genomic substitution rates in order to perform the substitution rate correction (see above) to prevent potential biases introduced by fast or slow evolving groups.

### Simulations

We generated random sequences of 3,000 nucleotides-long and evolved these sequences following the species’ tree using GenSeq (version 1.3.4) (Rambaut and Grassly 1997). We used the same species used in the previous *d*_S_-based analyses. The species’ tree was downloaded from the Timetree database (http://www.timetree.org/; last accessed October 16, 2019). The squamate tree was reshaped based on ref. (Zheng and Wiens 2016). We transformed the resulting branch lengths (representing millions of years) to substitution rates using a value of 2.22e-09 mutations per million years, which is an averaged substitution rate derived from a wide number of mammalian genomes (Kumar and Subramanian 2002); a similar estimate is not yet available for pleurodonts; we considered the mammalian substitution rate as a proxy of the amniote substitution rate. We reckon that mutation rates may be affected by different variables such as extended generation times and late sexual maturity. Generation times among the selected reptiles, except for *A. carolinensis* that has a generation time of 1 year, are similar: 2–3.5 years in agamids and pleurodonts, and 3–4 years in snakes. We modeled nucleotide evolution under 16 scenarios. Under the first scenario, the null hypothesis, agamids never had the XY chromosomes, their branches were not affected by MMB and their lengths were equivalent to those from snakes. In this null scenario, sequences from snakes and agamids were evolving at a rate of 2.22e-09 mutations per million years. Then, we modeled nucleotide evolution under 15 alternative scenarios under four different strengths of α, 1.8 (α reported in snakes; Vicoso et al. 2013), 2.4 (α in most mammals −2.4 ± 0.26 95% CI—Wilson Sayres et al. 2011; Link et al. 2017 and birds −2.47 ± 0.2 95% CI—Bartosch-Harlid et al. 2003), 4, and 6 (values of α reported for primates; Makova and Li 2002; Venn et al. 2014 and pleurodonts; this study). GenSeq (Rambaut and Grassly 1997) uses two different models (GTR and HKY) and, based on the branch lengths, it applies a particular substitution rate. Thus, by modifying the branch lengths, we were able to simulate the effect of MMB. For each of the 15 alternative scenarios, we gradually shortened the branch lengths in the agamid clade (or in specific branches) by 0.00683, 0.0091, 0.0151, and 0.0227, which represented the substitution rates occurring over a period of 10 Myr under α values of 18, 2.4, 4, and 6, respectively. The higher the strength of MMB, the more rapidly the branch lengths will be reduced. In other words, by shortening the branch lengths of agamids by the indicated values, the species in this lineage were no longer evolving at a rate of 2.22e-09 mutations per million years for 170 Myr (approximate age of the sex chromosomes), and the differences in lengths were equivalent to the substitution rate owing to MMB during the amount of time the sequences were X-linked. GenSeq (Rambaut and Grassly 1997) is a high-speed program and it can be run with multiple processes in parallel. We repeated the process with 100 different randomly generated nucleotide sequences for each simulation. During the simulations, snake sequences were not differentially affected by MMB, whereas pleurodont sequences were always affected by α = 6. Finally, for each set of evolved sequences, we calculated a *d*_S_ tree using the codeml program, implemented in the PAML44 package (Yang 1997).

For placental mammals, we performed simulations as described above with few changes. The species’ tree was downloaded from the Timetree database (http://www.timetree.org/; last accessed October 16, 2019). We transformed the resulting branch lengths (representing millions of years) to substitution rates using a value of 2.22e-09 mutations per million years, which is an averaged substitution rate derived from a wide number of mammalian genomes (Kumar and Subramanian 2002). Prior to the simulations, we modified the branch lengths on the tree to simulate the effect of MMB in placental mammals reported in ref. (Wilson Sayres et al. 2011). That is, we shortened the branch lengths in the placental clade by 0.122 when α = 1.6–2 (rat, dog, and armadillo); by 0.198 when α = 2.9–3.3 (rabbit and elephant); by 0.27 when α = 3.9–4 (dolphin); by 0.39 when α >6 (human). The internal nodes were modified following the tree structure and averaging the α values of the tips or the previous branches. We modeled nucleotide evolution under 18 scenarios. Under the first scenario, the null hypothesis, platypus never had the placental XY chromosomes and its branch was not affected by MMB and the length was equivalent to those from the outgroup species. Then, we modeled nucleotide evolution under 17 alternative scenarios; two additional scenarios compared to reptiles since the placental sex chromosomes are 10–20 Myr older than the sex chromosomes in pleurodonts/acrodonts (Cortez et al. 2014; Marin et al. 2017). In these alternative scenarios, we considered that platypus shared the placental XY chromosomes for increasing periods of time. Thus, platypus’ branch was shortened by 0.0113 every 10 Myr based on the averaged α value of 2.9 reported in ref. (Link et al. 2017) for monotreme mammals. Finally, we ran 100 times each hypothesis using a different set of randomly generated sequences and every time we calculated a *d*_S_ tree using the codeml program, implemented in the PAML44 package (Yang 1997).

**Fig. 2.**
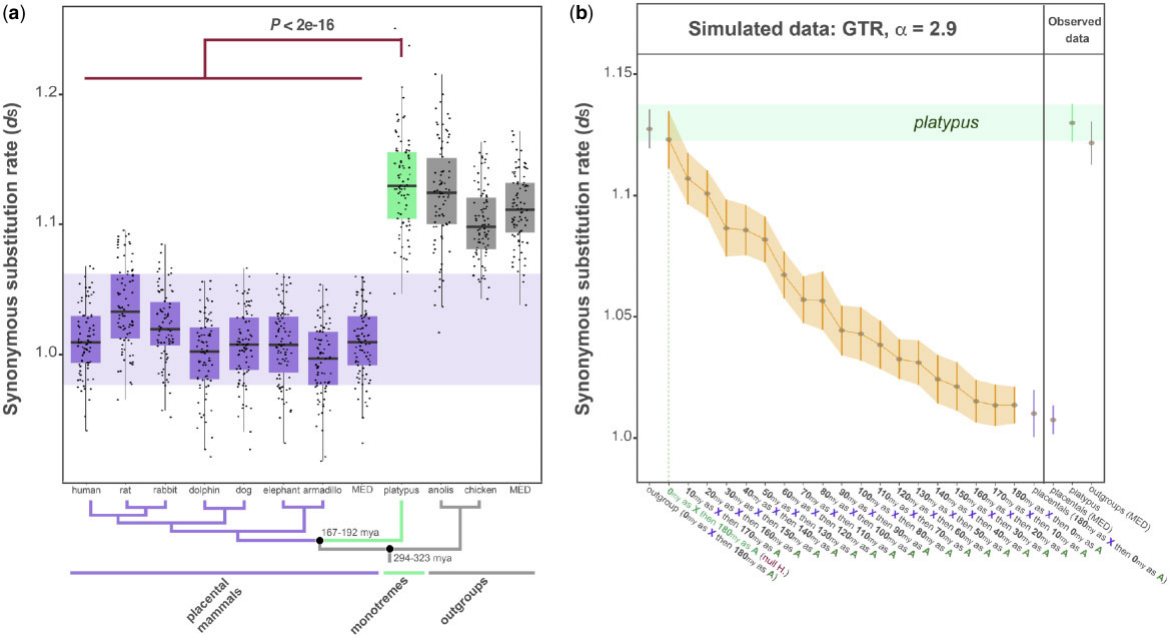
—Synonymous substitution rates of X-linked genes in placental mammals and orthologous genes in platypus and outgroup species. (*a*) Boxplots of autosomal-corrected synonymous substitution rates of X-linked sequences in placental mammals and orthologous sequences in platypus and outgroup species obtained from the concatenated alignment of 114 genes analyzed by 100 bootstrap rounds (see Materials and Methods). MED indicates the median value within placental or outgroup species. The shaded purple area marks values covered by placental mammals. Benjamini–Hochberg-corrected *P* values, Welch two sample *t*-tests. Error bars, maximum and minimum values, excluding outliers. Black dots represent the values from 100 bootstrap rounds. The species tree specifies the relationships among the analyzed species. (*b*) Platypus sequences were simulated as X-linked for different amounts of time and under α = 2.9 (see Materials and Methods). In the first scenario (the null hypotheses; red label), platypus sequences were modeled as X-linked sequences for 0 Myr and then modeled as autosomal sequences for 180 Myr. In the 17 alternative scenarios, platypus sequences were modeled as X-linked sequences for an increasing number of millions of years (by steps of 10 Myr) and then modeled as autosomal sequences for a decreasing number of millions of years (by steps of 10 Myr). Outgroup sequences were evolved in the absence of male mutation bias, whereas placental sequences were always evolved under specific α values (see Materials and Methods). MED indicates the median value within placental or outgroup species. Error bars indicate the Welch’s 95% confident intervals and the brown dots represent the mean values from 100 simulations. The shaded orange area highlights the pattern followed by the simulated data. The potential ages when the XY chromosome system loss would occur is given by the overlap between the observed and the simulated data (green horizontal bar). The overlap is also highlighted by the green dotted vertical line and the colored label on the *x* axis (light green).

The values plotted in figures 2, 6, and 5, and supplementary figures 1 and 2, Supplementary Material online, were taken from the *d*_S_ trees. All simulations were run using balanced compositions of nucleotides due to the specificities of GenSeq (Rambaut and Grassly 1997). Therefore, when plotting the data we adjusted the scale of the simulated data to match the scale of the observed data to account for changes in GC content in the species’ sequences.

### Statistical Analyses and Graphics

Statistical tests were performed using the R package, standard libraries. Data were plotted using the R package, “ggplot2” library.

## Results

### Monotreme Mammals Did Not Share the Same XY Chromosome System with Placental Mammals

We assessed the evolution at neutral sites using branch-specific rates of synonymous substitutions (*d*_S_ trees) from 114 genes that are X-linked in placental mammals but are autosomal in platypus and outgroup species. We generated 100 bootstrapped *d*_S_ trees based on the concatenated alignment of the 114 X-linked genes and one-to-one orthologous sequences in platypus and outgroup species. To correct for potential biases introduced by fast or slow evolving lineages, we corrected the X-linked substitution rates using autosomal substitution rates (see Materials and Methods), which can be used as proxies of the overall genomic mutation rate in each lineage. Figure 2*a* summarizes the autosomal-corrected X-linked substitution rates. We observed that platypus and the outgroup species showed similar values significantly higher compared to the substitution rates exhibited by placental mammals.

Finally, we ran simulations (see Materials and Methods) using a set of randomly generated sequences, the GTR substitution model, following the species’ tree, and using the known (Wilson Sayres et al. 2011; Link et al. 2017) male mutation bias intensities (α values) for each group. Each simulation tested a specific scenario in which platypus maintained the placental sex chromosomes for an increasing number of million years. We then compared the simulated and the observed data (from fig. 2*b*). We found that the observed data overlapped the simulated data only when the tested scenario specified that platypus never shared the placental XY chromosome system (fig. 2*c*). These results strongly indicate that platypus most likely did not share the sex chromosomes present in placental mammals.

### Male Mutation Bias in the Green Anole and the Fiji-Banded Iguana

**Fig. 3.**
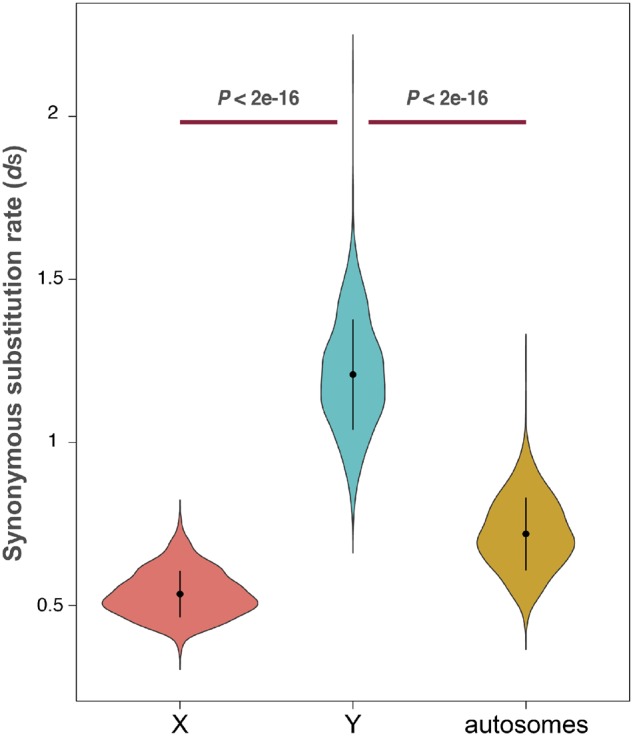
—Synonymous substitution rates for sex-linked and autosomal sequences in pleurodonts. Violin plots of synonymous substitution rates obtained from the comparison of Y, X, and autosomal intronic sequences between two pleurodont species, the green anole *Anolis carolinenesis* and the Fiji-banded iguana. *P* value, Welch two sample *t*-test. *N* = 1,000 bootstrap rounds. Error bars represent 1 SD from the mean.

In order to apply the same approach in squamates, we first needed to determine whether these species presented male mutation bias. Therefore, we analyzed the substitution rates at neutral sites of intronic sequences from X-linked, Y-linked, and autosomal genes between two pleurodont species, *A. carolinenesis* and the Fiji-banded iguana (*B. fasciatus*), and found that Y-linked sequences have evolved faster than both the X-linked and autosomal sequences (Welch two sample *t*-test, *P *<* *2e-16; fig. 3). The observed pattern is consistent with the presence of MMB (Link et al. 2017) in squamates. We applied Miyata’s equations (Miyata et al. 1987) to the median values of X, Y, and autosomal substitution rates and calculated the strength of MMB (α) in pleurodonts. We obtained an α value of 6 (95% confident intervals: 5.7–7) for the Y/X comparison, an α value of 5 (95% confident intervals: 3.2–7) for the X/A comparison, and an α value of 7.5 (95% confident intervals: 7–11) for the Y/A comparison, corresponding to a strong MMB in pleurodonts. The α values are similar and the confident intervals overlap in the three comparisons, which is consistent with MMB being the primary force shaping the chromosomal substitution rates (Link et al. 2017) in these species.

### Substitution Rates in X-Linked Sequences in Pleurodonts and Orthologous Genes in Agamids and Snakes

**Fig. 4.**
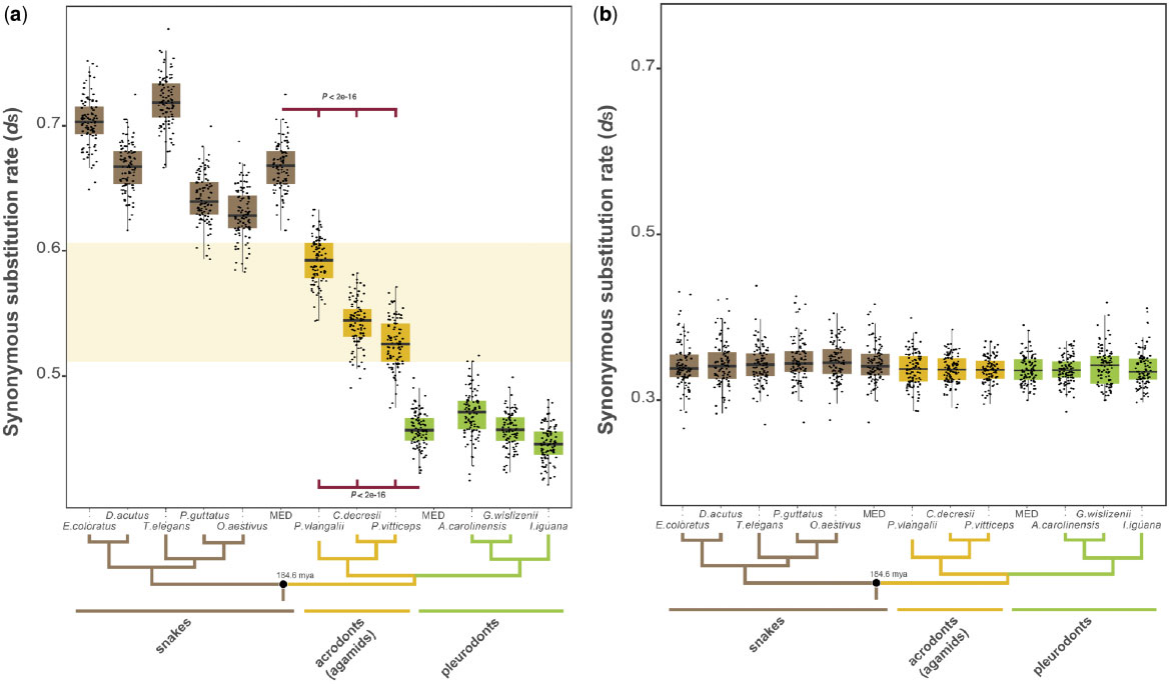
—Synonymous substitution rates of X-linked genes and autosomes in pleurodonts and orthologous genes in agamids and snakes. (*a*) Boxplots of autosomal-corrected synonymous substitution rates for X-linked sequences in pleurodonts and orthologous sequences in acrodonts (agamids) and snakes obtained from the concatenated alignment of 77 genes analyzed by 100 bootstrap rounds (see Materials and Methods). MED indicates the median value within placental or outgroup species. The shaded yellow area marks values covered by agamids. Benjamini–Hochberg-corrected *P* values, Welch two sample *t*-tests. Error bars, maximum and minimum values, excluding outliers. Black dots represent the values from 100 bootstrap rounds. The species tree specifies the relationships among the analyzed species. (*c*) Same as in (*b*) but for autosomal-corrected synonymous substitution rates of random subsets of autosomal sequences.

We estimated the substitution rates at neutral sites across distant taxa by building *d*_S_ trees with the coding sequences of X-linked genes in pleurodonts and one-to-one orthologous sequences in agamids, snakes, and other amniote species (see Materials and Methods). We produced 100 individual bootstrapping *d*_S_ trees based on the concatenated alignment of the 77 X-linked genes and one-to-one orthologous sequences in other species. We corrected the X-linked substitution rates using autosomal substitution rates to reduce the effect of fast or slow evolving lineages. The results indicated that the snake orthologous sequences (i.e., sequences that were not X-linked) have evolved faster than the sequences from pleurodonts (i.e., sequences that have been X-linked for 170–160 Myr; Benjamini–Hochberg corrected *P *<* *0.001, Welch two sample *t*-test; fig. 4*a* and *b*). Importantly, the sequences from the three species of agamids (*P. vitticeps*, *C. decresii*, and *Phr.vlangalii*) showed branch lengths that were significantly larger than those from pleurodonts, but significantly shorter than those from snakes (Benjamini–Hochberg corrected *P *<* *0.001, Welch two sample *t*-test; fig. 4*a* and *b*). We could not find significant differences when random subsets of autosomal genes were taken as a substitute for the X-linked genes (Benjamini–Hochberg corrected *P *>* *0.05, Welch two sample *t*-test; fig. 4*c*). These results were consistent with the presence of male mutation bias in agamids and strongly supported the hypothesis that agamids shared the XY chromosome system with pleurodonts during a number of million years. Lastly, the clear differences in substitution rates between *P. vitticeps/C. decresii* and *Phr.vlangalii* could indicate that the XY chromosome system was conserved longer in the *Pogona/Ctenophorus* lineage or, alternatively, that the *Pogona/Ctenophorus* and *Phrynocephalus* lineages experienced different strengths of MMB.

### Simulated Data in Squamates

**Fig. 5.**
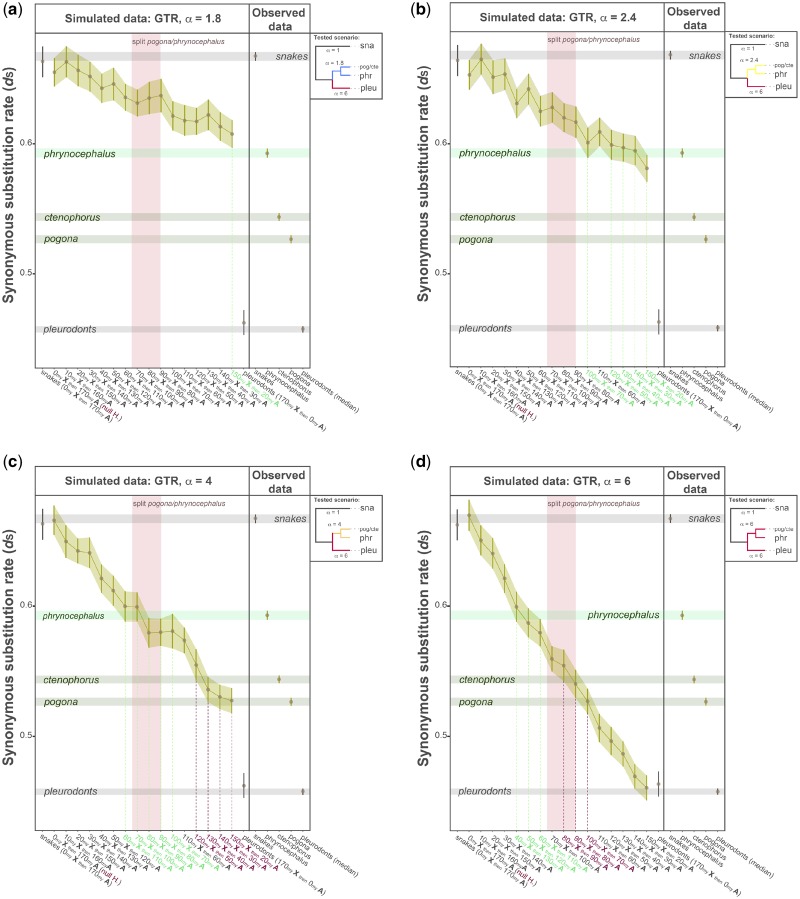
—Simulations in squamates using the GTR model. Agamid sequences were simulated as X-linked for different amounts of time. *Phrynocephalus* and *Pogona/Ctenophorus* lineages were subjected to constant intensities of α: (*a*) α = 1.8, (*b*) α = 2.4, (*c*) α = 4, and (*d*) α = 6. In the first scenario (the null hypotheses; red labels), agamid sequences were modeled as X-linked sequences for 0 Myr and then modeled as autosomal sequences for 170 Myr. In the 15 alternative scenarios, agamid sequences were modeled as X-linked sequences for an increasing number of millions of years (by steps of 10 Myr) and then modeled as autosomal sequences for a decreasing number of millions of years (by steps of 10 Myr). Snake sequences were evolved in the absence of male mutation bias, whereas pleurodont sequences were always evolved under strong male mutation bias (α = 6). The trees in the lateral boxes summarize the strength of α applied to the different groups; *sna* is snakes, *pog* is pogona, *cte* is ctenophorus, *phr* is phrynocephalus, and *pleu* are pleurodonts. Error bars indicate the Welch’s 95% confident intervals and the brown dots represent the mean values from 100 simulations. The green shaded areas highlight the patterns followed by the simulated data. The potential ages when the XY chromosome system loss would occur are given by the overlap between the observed and the simulated data (green horizontal bars, light and dark green for *Phrynocephalus* and *Pogona/Ctenophorus* lineages, respectively). These overlaps are also highlighted by the dotted vertical lines and the colored labels on the *x* axis (light green and magenta for *Phrynocephalus* and *Pogona/Ctenophorus* lineages, respectively). The pink vertical bars indicate the *Pogona/Phrynocephalus* speciation event (∼79 Myr after the last common ancestor of the two lineages; taken from Zheng and Wiens 2016).

**Fig. 6.**
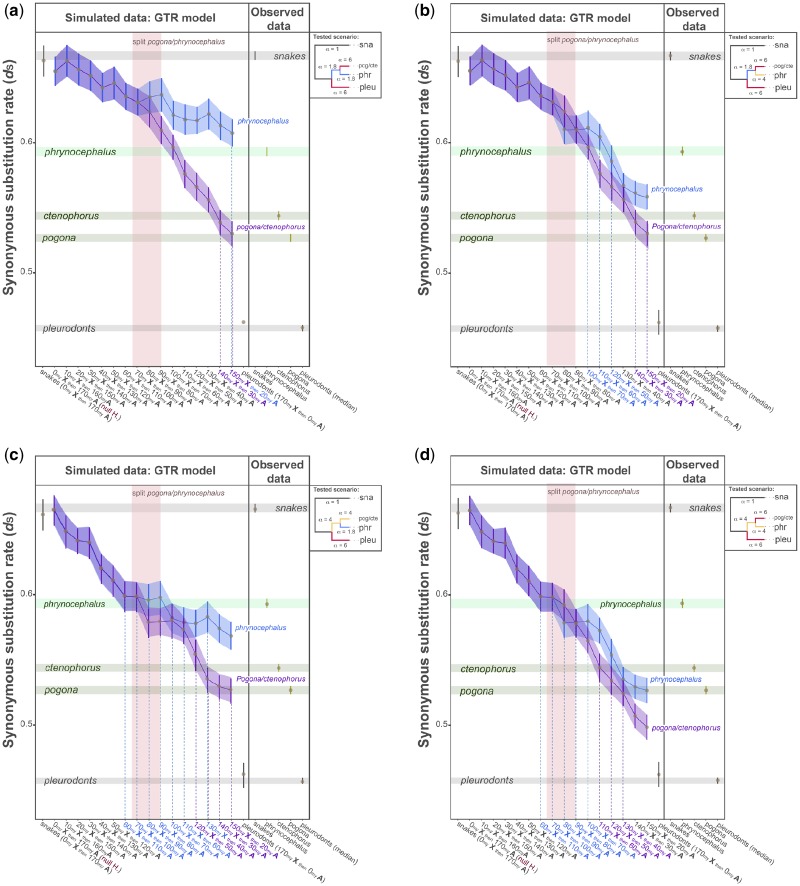
—Simulated data in squamates using the GTR model. Agamid sequences were simulated as X-linked for different amounts of time. *Phrynocephalus* and *Pogona/Ctenophorus* lineages were subjected to variable intensities of α: (a) low-strong, (b) low-medium-strong, (c) medium-low-strong, and (d) medium-strong. In the first scenario (the null hypotheses; red labels), agamid sequences were modeled as X-linked sequences for 0 Myr and then modeled as autosomal sequences for 170 Myr. In the 15 alternative scenarios, agamid sequences were modeled as X-linked sequences for an increasing number of millions of years (by steps of 10 Myr) and then modeled as autosomal sequences for a decreasing number of millions of years (by steps of 10 Myr). Snake sequences were evolved in the absence of male mutation bias, whereas pleurodont sequences were always evolved under strong male mutation bias (α = 6). The trees in the lateral boxes summarize the strength of α applied to the different groups; *sna* is snakes, *pog* is pogona, *cte* is ctenophorus, *phr* is phrynocephalus, and *pleu* are pleurodonts. Error bars indicate the Welch’s 95% confident intervals and the brown dots represent the mean values from 100 simulations. Shaded areas highlight the patterns followed by the simulated data (blue for *Phrynocephalus* and purple for *Pogona/Ctenophorus*). The potential ages when the XY chromosome system loss would occur are given by the overlap between the observed and the simulated data (green horizontal bars, light and dark green for *Phrynocephalus* and *Pogona/Ctenophorus* lineages, respectively). These overlaps are also highlighted by the dotted vertical lines and the colored labels on the *x* axis (blue and purple for *Phrynocephalus* and *Pogona/Ctenophorus* lineages, respectively). The pink vertical bars indicate the *Pogona/Phrynocephalus* speciation event (∼79 Myr after the last common ancestor of the two lineages; taken from Zheng and Wiens 2016).

We evolved nucleotide sequences considering 16 different scenarios. The first scenario (i.e., the null hypothesis; figs. 5 and 6, red labels) considered that agamids did not share the ancestral XY chromosome system with pleurodonts. In addition, we modeled 15 alternative scenarios in which we assumed that agamids maintained the ancestral XY chromosome system for an increasing number of million years (fig. 5 and supplementary fig. 1, Supplementary Material online; see Materials and Methods). Since α values are not available for agamids due to scarce sequencing data, we decided to use different intensities of male mutation bias (fig. 5 and supplementary fig. 1, Supplementary Material online). In the simulations, the branch lengths of snakes and pleurodonts were not different between the observed values and simulated values (Mann–Whitney *U* test, *P *>* *0.05; fig. 5 and supplementary fig. 1, Supplementary Material online, grey horizontal bars). This result indicates that the simulations can correctly recapitulate that autosomal sequences in snakes have not been differentially affected by MMB, whereas X-linked sequences in pleurodonts have been under a strong MMB. On the other hand, the longer the sequences in agamids were modeled as X-linked, and were differentially affected by MMB, their branch lengths became shorter, moved away from the snake values and approached the values exhibited by pleurodonts (fig. 5 and supplementary fig. 1, Supplementary Material online, shaded areas). Importantly, the simulations supported the hypothesis that agamids conserved the ancestral XY chromosome system for several million years. In other words, when we assumed that agamids did not share the ancestral XY chromosome system with pleurodonts (fig. 5 and supplementary fig. 1, Supplementary Material online, the red “*null H.*” label on the *x* axis), regardless of the intensity of MMB, the results were incompatible with the observed data for the three species (no overlap between the observed values and the simulated values; fig. 5 and supplementary fig. 1, Supplementary Material online, horizontal light and dark green bars).

We further explored the simulated data to infer the approximate time at which agamids lost the XY chromosome system. To do so, we matched the observed values and the simulated values for *Pogona/Ctenophorus* as a single lineage, given their close phylogenetic relationship (Zheng and Wiens 2016), and *Phrynocephalus* as another lineage (fig. 5 and supplementary fig. 1, Supplementary Material online, horizontal light and dark green bars). We initially assumed that these lineages were affected by similar intensities of MMB. The results obtained with low values of α (1.8 and 2.4) were incompatible with the observed data for *Pogona/Ctenophorus* (no overlap between the observed values and the simulated values; fig. 5*a* and *b* and supplementary fig. 1, Supplementary Material online). In addition, the results obtained using a high value of α (α = 6) implied that *Phrynocephalus* lost the XY chromosome system in the ancestor of both groups, but *Pogona/Ctenophorus* lost the XY chromosome system after the speciation event (fig. 5*d* and supplementary fig. 1, Supplementary Material online, vertical pink line), which would mean that *Pogona/Ctenophorus* lost the sex chromosomes twice. Only the simulations using a medium value of α (α= 4) provided sounded results. In this scenario, the *Pogona/Ctenophorus* lineage lost the XY chromosome system 20–50 Ma (fig. 5*c* and supplementary fig. 1, Supplementary Material online), whereas the *Phrynocephalus* lineage lost the XY chromosome system 70–110 Ma (fig. 5*c* and supplementary fig. 1, Supplementary Material online), which coincides with the time it diverged from *Pogona/Ctenophorus* (105.5  Ma; 79 Myr after the common ancestor of *Pogona* and *Phrynocephalus* lineages; Zheng and Wiens 2016).

It is, however, very likely that the *Pogona/Ctenophorus* and *Phrynocephalus* lineages experienced different intensities of MMB after their divergence. Changes in MMB intensities have been seen in placental mammals (Wilson Sayres et al. 2011) and have the potential to affect the results. Therefore, we explored combinations of low, medium, and high values of α, both in the ancestor and in the specific branches leading to *Pogona/Ctenophorus* and *Phrynocephalus*. In agreement with the first round of simulations (results in fig. 5), we found that a combination of low values of α (α < 4) were incompatible with the observed data in *Pogona/Ctenophorus*, and a combination of high values of α (α > 6) resulted in *Pogona/Ctenophorus* losing the XY chromosome system twice. Ultimately, only four scenarios were compatible with the observed data, as well as with the evolution of the species (i.e., divergence time; fig. 6 and supplementary fig. 2, Supplementary Material online). In the first scenario (fig. 6*a*), the ancestor and *Phrynocephalus* lineage experienced low MMB, but *Pogona/Ctenophorus* specifically experienced an acute increase in α. In this scenario, *Pogona/Ctenophorus* and *Phrynocephalus* lost the XY chromosome system very recently (20–30 Ma). Other plausible scenarios included (i) an independent increase of α in agamids (fig. 6*b*), (ii) an increase of α in *Pogona/Ctenophorus* coupled with a decrease of α in *Phrynocephalus* (fig. 6*c*), and (iii) an increase of α specifically in *Pogona/Ctenophorus* coupled with moderate α in *Phrynocephalus* (fig. 6*d*). These three scenarios provided similar results, meaning *Pogona/Ctenophorus* consistently lost the XY chromosome system in recent times, 20–60 Ma, whereas *Phrynocephalus* lost the XY chromosome system around the time the *Pogona* and *Phrynocephalus* lineages split (105.5 Ma; 79 Myr after the common ancestor of *Pogona* and *Phrynocephalus* lineages; Zheng and Wiens 2016).

## Discussion

Overall, our work showed that by tracing the effects of male mutation bias on a specific group of sequences we could hint at former X chromosomes. Specifically, we first showed that monotremes did not share the sex chromosomes with placental mammals. A number of studies support this result since phylogenetic analyses of both monotreme X_5_Y_5_ and therian XY gametologs indicated an independent origin for the sex chromosomes in these two groups (Cortez et al. 2014). Furthermore, we showed that pleurodonts and acrodonts probably shared the same sex chromosomes, and that acrodonts conserved the pleurodont XY chromosomes for several millions of years.

In the majority of the scenarios we tested, either with fixed or variable α values, we obtained similar results. That is, the *Pogona/Ctenophorus* lineage probably lost the XY chromosome system in more recent times than the *Phrynocephalus* lineage, which seemed to have probably lost the XY chromosome system around the time it diverged from *Pogona*. This result entails an interesting possibility since a sex determination system turnover could have been a key factor promoting the speciation of the *Phrynocephalus* lineage. At this stage, we cannot know which genetic or environmental factors triggered the sex chromosome turnover in acrodonts and whether this event responded to either selection pressures or genetic drift (Saunders et al. 2018).

So far, the Tree of Sex database contains information regarding the sex determination systems in 27 species of agamids and 3 species of chameleons (Bachtrog et al. 2014). Additional studies have added a few extra species to the list (Rovatsos et al. 2015; Hansson and Olsson 2018; Nielsen et al. 2018). However, there are ∼500 extant species in the acrodont clade. We should, therefore, continue with the characterization of the sex determination systems in this group since some species may have maintained the ancestral XY chromosome system we still observe in pleurodonts.

We note that the reported age estimates for the sex determination system turnovers in acrodonts should be taken with prudence. At this stage, our method could hint at whether a particular group of species shared specific sex chromosomes during an approximated range of time before undergoing a sex determination system turnover. We note, however, that although the general patterns are robust, the variation in synonymous substitution rates is high within groups, particularly in snakes. This could be due to species-specific variations in the mutation rates or the strength of MMB. But also due to the heterogeneity in synonymous substitution rates among autosomes of the same species. Therefore, we cannot exclude that all agamid groups lost the pleurodont XY system in a single ancestral event. Our results will surely be refined as we learn more about the mutation rates and X chromosome strata in reptiles. More accurate estimates will require knowing the strength of MMB in a larger number of species of pleurodonts, acrodonts, and snakes, and for this, further sequencing data will be required. However, simulations offer a good alternative since they present direct estimates that derive from the sole effect of MMB, regardless of any confounding factor.

The method described in this work could be suitable to study a broad number of groups where sex turnovers are suspected but the obvious genomic signatures have been eroded owing to the loss of the Y chromosome and the present autosomal nature of the former X chromosome. The method could also be applied in species with ZW chromosomes. Male mutation bias would make the Z chromosome to evolve faster because it spends more time in the male germline (Wilson Sayres and Makova 2011). The simulations should be modified accordingly. We note that the only prerequisites to apply the method are sufficient sequencing data and presence of MMB in the groups of interest. Snakes are a potential candidate group to test the method. One could test whether pythons and boas shared the canonical ZW system before evolving specific XY systems (Gamble et al. 2017; Augstenova et al. 2018). Another example could be amphibians and their recently described panoply of sex chromosome transitions (Jeffries et al. 2018). 

## Supplementary Material

Supplementary data are available at *Genome Biology and Evolution* online.

## Supplementary Material

evz221_Supplementary_Data

## Notes

**Data deposition:** This project has been deposited at NCBI-SRA database (www.ncbi.nlm.nih.gov/sra; last accessed October 16, 2019) under the accession BioProject PRJNA490698; RNAseq data for a female green iguana (*Iguana iguana*) and a female leopard-lizard (*Gambelia wislizenii*). Genomic sequences for a male *Anolis carolinensis* and a male Fiji-banded iguana (*Brachylophus fasciatus*) are available on the NCBI-SRA database, BioProject accession number PRJNA381064 (Marin et al. 2017). RNA-seq data for *Ctenophorus decresii*, *Phrynocephalus vlangalii*, *Deinagkistrodon acutus*, *Echis coloratus*, *Pantherophis guttatus*, and *Opheodrys aestivus* are available on the NCBI-SRA database, accession numbers PRJNA478483, PRJNA248542, PRJNA314559, and SRR1287707–SRR1287715, respectively (Hargreaves et al. 2014; Yang et al. 2014; Yin et al. 2016). RNA-seq data for *Thamnophis elegans* are available on the Reptilian Transcriptomes v2.0 database (Tzika et al. 2015). CDS sequences for *Anolis*, human, mouse, elephant, opossum, platypus, chicken, Chinese soft-shell turtle, and *Xenopus* are available on the ENSEMBL database (www.ensembl.org; last accessed October 16, 2019). CDS sequences for *Pogona vitticeps* are available on the Pogona Genome Project (Georges et al. 2015). Supplementary table 2, Supplementary Material online, summarizes the data used in this study.
