# Supplementary material for: Deciphering Ancestral Sex Chromosome Turnovers Based on Analysis of Male Mutation Bias
Source: Genome Biol Evol. 2019 Oct 12;11(11):3054–67. doi: 10.1093/gbe/evz221 (PMC6823514; doi:10.1093/gbe/evz221)
Supplement: evz221_Supplementary_Data [file evz221_supplementary_data.zip › Supplementary.Figures1-2.pdf]

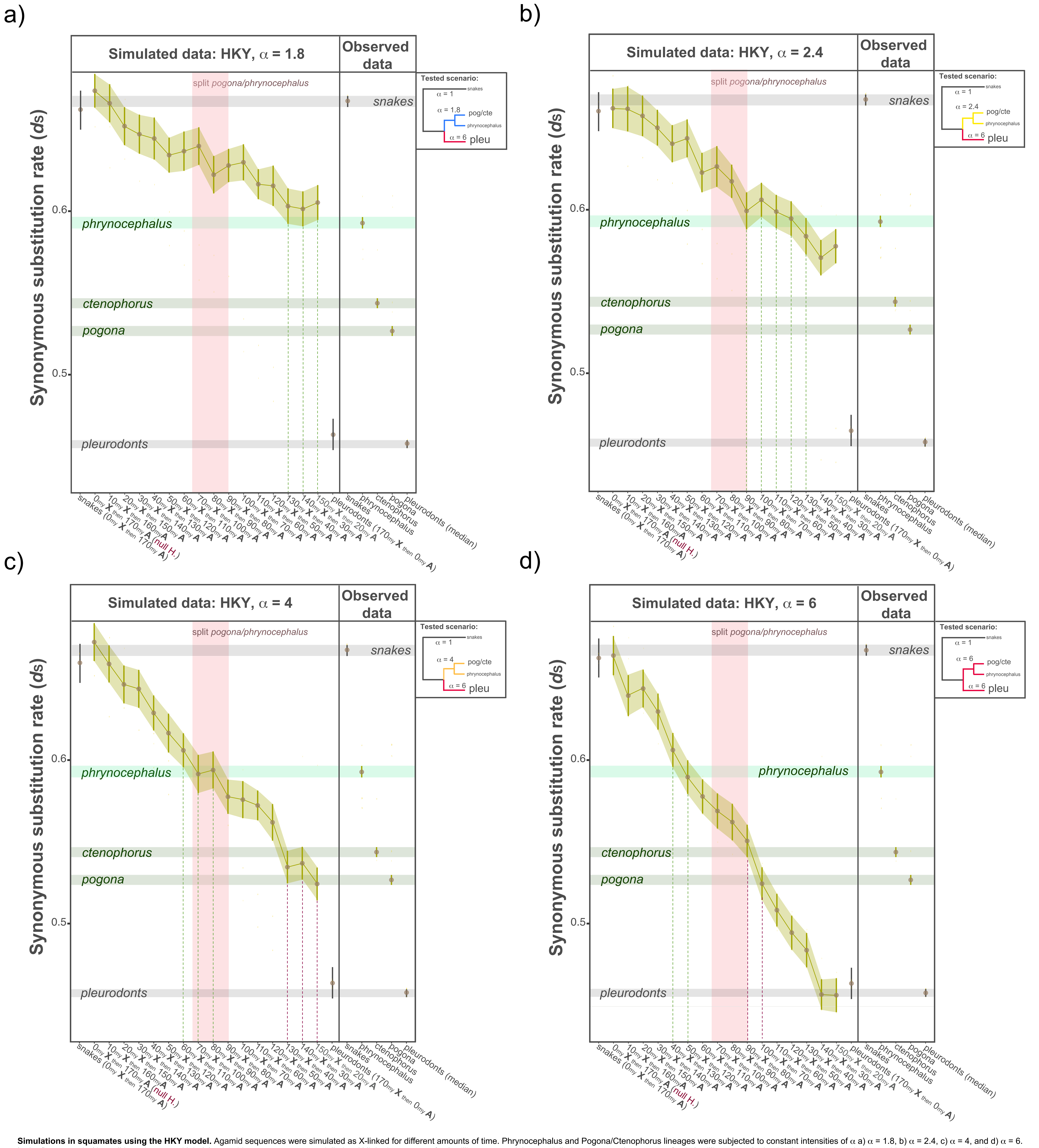

Simulations in squamates using the HKY model. Agamid sequences were simulated as X-linked for different amounts of time. Phrynocephalus and Pogona/Ctenophorus lineages were subjected to constant intensities of α a) α = 1.8, b) α = 2.4, c) α = 4, and d) α = 6. In the first scenario (the null hypotheses; red labels), agamid sequences were modelled as X-linked sequences for 0 million years and then modelled as autosomal sequences for 170 million years. In the 15 alternative scenarios, agamid sequences were modelled as X-linked sequences for an increasing number of millions of years (by steps of 10 million years) and then modelled as autosomal sequences for a decreasing number of millions of years (by steps of 10 million years). Snake sequences were evolved in the absence of male mutation bias, whereas pleurodont sequences were always evolved under strong male mutation bias (α = 6). The trees in the lateral boxes summarize the strength of α applied to the different groups; sna is snakes, pog is pogona, cte is ctenophorus, phr is phrynocephalus and pleu are pleurodonts. Error bars indicate the Welch's 95% confident intervals and the brown dots represent the mean values from 100 simulations. The green shaded areas highlight the patterns followed by the simulated data. The potential ages when the XY chromosome system loss would occur are given by the overlap between the observed and the simulated data (green horizontal bars, light and dark green for Phrynocephalus and Pogona/Ctenophorus lineages, respectively). These overlaps are also highlighted by the dotted vertical lines and the coloured labels on the X-axis (light green and magenta for Phrynocephalus and Pogona/Ctenophorus lineages, respectively). The pink vertical bars indicate the Pogona/Phrynocephalus speciation event (around 79 million years after the last common ancestor of the two lineages; taken from Zheng et al. 2016. Mol Phylogenet Evol.

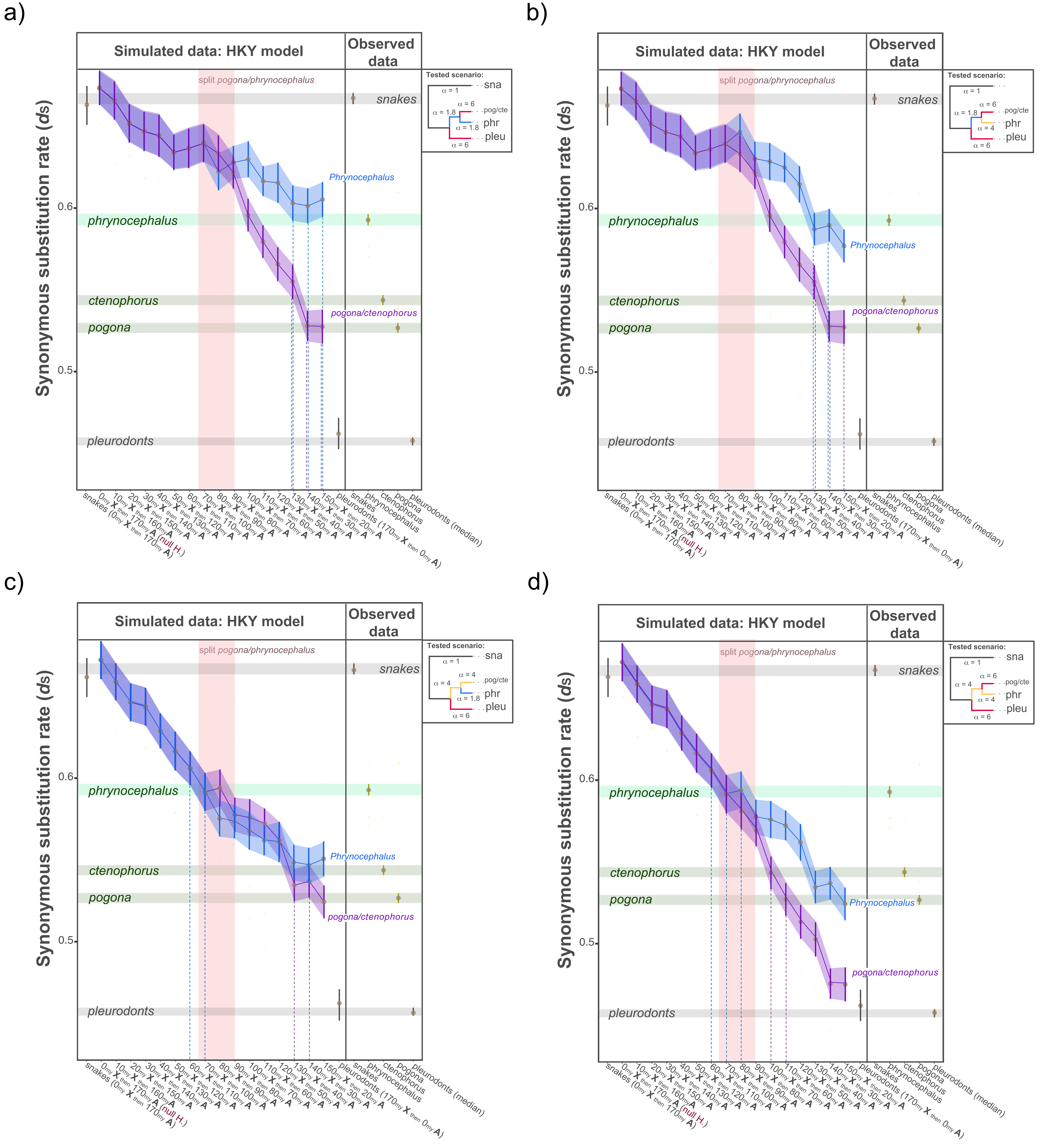

Simulated data in squamates using the HKY model. Agamid sequences were simulated as X-linked for different amounts of time. Phrynocephalus and Pogona/Ctenophorus lineages were subjected to variable intensities of α In the first scenario (the null hypotheses; red labels), agamid sequences were modelled as X-linked sequences for 0 million years and then modelled as autosomal sequences for 170 million years. In the 15 alternative scenarios, agamid sequences were modelled as X-linked sequences for an increasing number of millions of years (by steps of 10 million years) and then modelled as autosomal sequences for a decreasing number of millions of years (by steps of 10 million years). Snake sequences were evolved in the absence of male mutation bias, whereas pleurodont sequences were always evolved under strong male mutation bias (α = 6). The trees in the lateral boxes summarize the strength of α applied to the different groups; sna is snakes, pog is pogona, cte is ctenophorus, phr is phrynocephalus and pleu are pleurodonts. Error bars indicate the Welch's 95% confident intervals and the brown dots represent the mean values from 100 simulations. Shaded areas highlight the patterns followed by the simulated data (blue for Phrynocephalus and purple for Pogona/Ctenophorus). The potential ages when the XY chromosome system loss would occur are given by the overlap between the observed and the simulated data (green horizontal bars, light and dark green for Phrynocephalus and Pogona/Ctenophorus lineages, respectively). These overlaps are also highlighted by the dotted vertical lines and the coloured labels on the X-axis (blue and purple for Phrynocephalus and Pogona/Ctenophorus lineages, respectively). The pink vertical bars indicate the Pogona/Phrynocephalus speciation event (around 79 million years after the last common ancestor of the two lineages; taken from Zheng et al. 2016. Mol Phylogenet Evol.
